# Crossing the Cultural and Value Divide Between Health and Social Care

**DOI:** 10.5334/ijic.2534

**Published:** 2016-10-28

**Authors:** Robin Miller

**Affiliations:** Health Services Management Centre, University of Birmingham, Park House 40 Edgbaston Park Road, Birmingham, B15 2RT, GB

## Introduction

Collaboration between health and social care services is vital if we are to truly provide integrated care. Whilst structural barriers based on policy frameworks, resource availability and organisational difference contributes to fragmentation between these sectors, it is the clashes in organisational cultures and values that can often be the most divisive. Only by being honest about our own values and the underlying assumptions that they reflect, and by being willing to challenge ourselves and others about unhelpful cultures that have developed can we ensure integration that draws successfully on both sectors.

Since qualifying as a social worker I have spent over two decades practicing, managing, purchasing and researching care services. The exact nature of this diverse and evolving sector and indeed the use of the term ‘social care’ itself varies between country and continent. It commonly includes the provision of personal care and domestic support to enable people to maintain their independence or recover from a physical or mental trauma, and services that connect excluded people with wider community resources such as housing, education and employment. Most of my career has been in the context of care services which work closely with those in health, and this has convinced me that neither sector can fulfil its responsibilities or indeed achieve the aspiration of integrated care unless they collaborate successfully. In this perspective I will reflect on my personal experience and the wider evidence regarding health and social care integration, and the central role that culture and values play within this.

## Structural divides

The structural issues that commonly fragment health care services such as multiple organisations focused on discrete elements of a pathway, poor communication between patient record systems, and conflicting incentive and performance frameworks are also experienced between health and social care. In addition to these there are wider systemic differences which provide further divisions. The ‘social care’ sector incorporates a wide variety of services which seek to support people in improving their independence and social connections – training, employment, rehabilitation, carers support, safeguarding, personal care, statutory mental health assessment are but a few of the roles commonly provided. Such diversity is undoubtedly one of the sector’s strengths, but can make it challenging for those working in health to gain a grasp of what actually is ‘social care’. The organisations that deliver such services range from small volunteer run entities that work in a single locality and rely on charitable donations of time and money, up to national or indeed international social enterprises that draw on multiple and sophisticated funding streams. Again for those working in health care it can be bewildering trying to understand how such organisations are led, governed and organised. Finally there is also the issue of resources, and the reality that health services (and in particular clinical services) are generally better funded and their professionals more handsomely rewarded than those in social care. Differentials in service resourcing and individual salaries are of course found within the sectors, but social care often feels (and indeed is) the poorer cousin. Academic practice further reflects the wider resourcing and interest in health, with the numerous journals and funding streams relating to health care vastly outweighing those focussing on social care.

## Cultural divides

Organisational and professional cultures are a recurrent feature in research regarding the integration of services (see [1] for overview). Defined by Schneider and Barbera [2] as ‘the values and beliefs that characterize organizations, as transmitted by socialization processes that newcomers have, the decisions made by management, and the stories and myths people tell and retell about their organizations’ (p. 10), culture can be deeply held and have considerable influence on how services operate. I remember as a young social worker posted out to a community mental health team one of the few issues that brought together my health colleagues was a common frustration with ‘social services’. I was commonly referred to as the ‘one good social worker’, and to my eternal shame failed to challenge this blanket negativity as I sought individual acceptance from my more experienced health colleagues. Still today if you get a group of health professionals together there will be some who question how long it takes social care to make a decision, or the legitimacy of alternatively qualified practitioners to challenge their established professional perspectives (see e.g. [3]). Similarly it is common within adult social care strategy circles to portray health care management in derogatory terms – ‘being stitched up by health’ was and is a term I frequently hear used in strategic resource discussions, and I lose count of the number of times I have listened to social workers accusing medical receptionists of being obstructive. Both sides of the fence often fail to understand the other’s world, and even those who have many years of practice find it difficult to provide an accurate account of the pressures and processes experienced by colleagues in the other sector. The negativity can also be related to the classic work of Bion [4] and others in relation to group dynamics, and in particular the tendency for groups to blame ‘another’ for the anxieties they may feel about performing their own duties. This leads to a paralysis from the group as they fail then to recognise their own ability to address the problem in question.

## Value divides

Schein’s famous cultural model identifies the importance of values to understanding both the expressed and implicit elements of organisational patterns of behaviour [5]. Similar to culture, values are a strong underlying current that shapes our behaviour as individuals and as practitioners, with our value set being an amalgamation of personal, societal and professional influences. Alignment of values is recognised as a key component of successful organisational change, with perceived clashes being a source of resistance and disengagement from the process [6]. There is undoubtedly much in the values of health and social care sector that bring them together, with both sharing commitments to act in the best interests of the individual concerned, to be honest and transparent in communication, and challenge examples of poor care. However many studies report a clash in values between the two sectors as being an inhibitor of more integrated care. Reflecting Schein, in my view this is because there is often a difference between what we publically articulate in partnership settings (our *expressed values*) and what we actually think and feel in practice (our *implicit values*). The latter may include more negative beliefs, including the desire to uphold the role and status of our profession and to ensure that our professional perspective has a dominant position. This can lead resistance to collaborative working practices which may be perceived to result in our profession becoming less distinctive, even though in our heart we know that it is the right thing to do. Again it is common for practitioners and indeed senior managers to negatively label the values of the other sector in times of conflict as a means of avoiding more constructive engagement.

## Crossing the divide

Despite all these structural, cultural and values divides there are many inspiring examples of health and social care working well together. This is often found within services for excluded populations facing particular hardships such as the homeless or people with a learning disability. The vocational calling felt by many working in such services commonly results in a long-term commitment which enables sustained exposure to similarly minded colleagues in other sectors and the development of more open and trust based relationships. It is much harder within the hustle and bustle of mainstream health and social care services to gain such rich individual relationships, and targeted activities therefore need to be invested in. Information sessions, resource directories, service visits, referral protocols are all important ingredients of a more integrated health and social care system. But in my book they are not enough to address the issues above– we need to be braver in expressing the underlying tensions between the sectors, flush out and address the stereotypes, and seek opportunities to constructively and honestly engage with the other. Inter-professional learning can be a good means to facilitate openness and enquiry in a safe setting, with the opportunity to work on real life problems being a key motivator. Well managed teams can also provide the structure and processes to positively embrace professional diversities and so facilitate contribution from all. But ultimately all such opportunities require each of us within health and social care to accept our individual responsibilities, to reflect on and where necessary confront our own values, and to be a positive contributor to a more collaborative culture even if this means going against existing cultures and traditions.

## Author Information

Dr Robin Miller is a Senior Fellow and Director of Evaluation at the Health Services Management Centre and Co-Editor of the Journal of Integrated Care. He leads on a variety of applied research projects with a particular focus on evaluating and learning from change initiatives, and teaches regularly on post-graduate and leadership development courses.
